# Bacterial Cell Wall Analogue Peptides Control the Oligomeric States and Activity of the Glycopeptide Antibiotic Eremomycin: Solution NMR and Antimicrobial Studies

**DOI:** 10.3390/ph14020083

**Published:** 2021-01-22

**Authors:** László Izsépi, Réka Erdei, Anna N. Tevyashova, Natalia E. Grammatikova, Andrey E. Shchekotikhin, Pál Herczegh, Gyula Batta

**Affiliations:** 1 Doctoral School of Chemistry, University of Debrecen, H-4032 Debrecen, Egyetem tér 1., Hungary; izsepi.laszlo@gmail.com; 2 Department of Organic Chemistry, University of Debrecen, H-4032 Debrecen, Egyetem tér 1., Hungary; rerdeii@yahoo.com; 3 Gause Institute of New Antibiotics, 11 B. Pirogovskaya, 119021 Moscow, Russia; chulis@mail.ru (A.N.T.); ngrammatikova@yandex.ru (N.E.G.); shchekotikhin@mail.ru (A.E.S.); 4 Department of Pharmaceutical Chemistry, University of Debrecen, H-4032 Debrecen, Egyetem tér 1., Hungary; herczegh.pal@pharm.unideb.hu

**Keywords:** glycopeptide, resistance, eremomycin, dimer, oligomer, *N*-Ac-d-Ala-d-Ala, ligand, ^15^N relaxation, diffusion, NMR

## Abstract

For some time, glycopeptide antibiotics have been considered the last line of defense against Methicillin-resistant *Staphylococcus aureus* (MRSA). However, vancomycin resistance of Gram-positive bacteria is an increasingly emerging worldwide health problem. The mode of action of glycopeptide antibiotics is essentially the binding of peptidoglycan cell-wall fragments terminating in the d-Ala-d-Ala sequence to the carboxylate anion binding pocket of the antibiotic. Dimerization of these antibiotics in aqueous solution was shown to persist and even to enhance the antibacterial effect in a co-operative manner. Some works based on solid state (ss) Nuclear Magnetic Resonance (NMR) studies questioned the presence of dimers under the conditions of ssNMR while in a few cases, higher-order oligomers associated with contiguous back-to-back and face-to-face dimers were observed in the crystal phase. However, it is not proved if such oligomers persist in aqueous solutions. With the aid of ^15^N-labelled eremomycin using ^15^N relaxation and diffusion NMR methods, we observed tetramers and octamers when the *N*-Ac-d-Ala-d-Ala dipeptide was added. To the contrary, the *N*-Ac-d-Ala or (*N*-Ac)_2_-l-Lys-d-Ala-d-Ala tripeptide did not induce higher-order oligomers. These observations are interesting examples of tailored supramolecular self-organization. New antimicrobial tests have also been carried out with these self-assemblies against MRSA and VRE (resistant) strains.

## 1. Introduction

If the fear that infections by both Methicillin-resistant *Staphylococcus aureus* (MRSA) and vancomycin-resistant *enterococci* (VRE) bacteria will spread and cause worldwide epidemics becomes true, we may return to the pre-penicillin age in which bacterial infection contributed significantly to morbidity rates. Vancomycin-resistant bacteria have adopted a simple, but powerful device for their survival: the essential d-Ala-d-Ala target sequence normally found in vancomycin-sensitive bacteria has changed to d-Ala-d-Lac. Experimental and theoretical studies have shown that vancomycin binds less strongly to such targets [1]. The newer VanC type resistance arises from the sixfold-lower affinity of vancomycin for acyl-d-Ala-d-Ser than for acyl-d-Ala-d-Ala [2]. There is an ongoing interest in the modification of glycopeptide antibiotics [3] and search for new antibiotics [4]. An in-depth review of the structural biology of molecular recognition by vancomycin antibiotics was published [5]. It is known that most vancomycin antibiotics exert an enhanced cell-wall analogue ligand binding through the formation of back-to-back dimers [6,7,8,9,10,11,12,13] in aqueous solution. 

The non-covalent dimers persist in both the presence and absence of small peptide ligands. Bulk methods, such as microcalorimetry [14] and sedimentation equilibrium [15], should provide valuable details on the thermodynamics of the aggregates. However, using these methods, it is difficult to differentiate between dimers and possible higher-order oligomers. Thermal titration curves of the association between vancomycin and peptides suggested ligand- induced aggregation, inconsistent with a simple 1:1 stoichiometry [14]. X-ray studies of vancomycin [16] and balhimycin [17] indicate the presence of four copies of antibiotic molecules in an asymmetric unit of the crystal. 

These molecules form the known antiparallel back-to-back dimers [9]. However, a face-to-face dimer interface is also observed, suggesting the formation of a virtually infinite chain via inter-dimeric H-bonds. In the crystal phase, such a structure was observed with a small ligand, *N*-Ac-d-Ala [16]. Theoretically, larger ligands with different stoichiometry may also induce higher-order oligomers. It was suggested [5,16], that the antibiotic/peptide stoichiometry may be 1:1 or 2:1 depending on the type of the ligand. From X-ray studies, a putative model suggests that it is possible to detect ligand-mediated and dependent oligomerization in solution, when face-face dimers are formed, and a docking site for a third dimer is created that stabilizes the face-to-face interaction [18,19]. 

The crystal asymmetric unit contains six monomers [20] of vancomycin and similarly of balhimycin [21]. Other biophysical methods (SAXS, DLS) supported the presence of vancomycin- complexed *N*-Ac-d-Ala-d-Ala hexamers. In fact, surface plasmon resonance studies [22,23] generally reveal better correlation between the cell-wall analogue peptide binding constant and antimicrobial activity. Interestingly, inhibition of the activity of eremomycin with an access tripeptide (*N*-Ac)_2_-l-Lys-d-Ala-d-Ala required 10–50 times higher concentration than for vancomycin in competition experiments [24]. 

**Figure 1:**
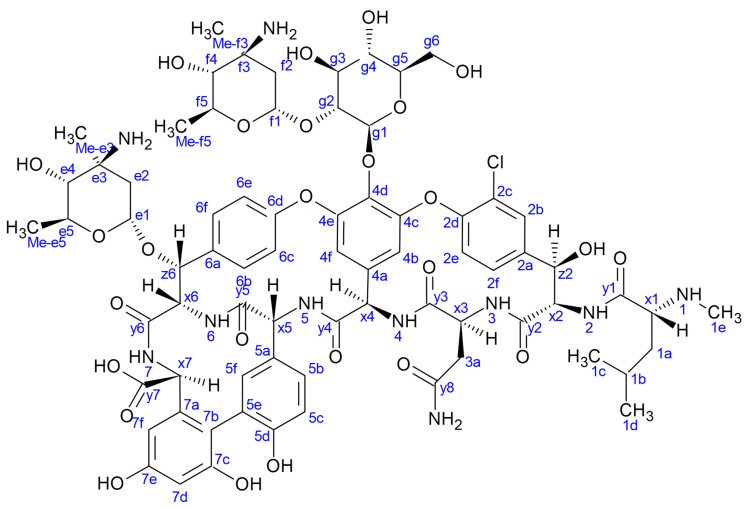
The structure of eremomycin. Labels **e** and **f** indicate eremosamine; **g** indicates glucopyranose sugar units. Numbering accord to amino acid sequential order, **x** stand for α-CHs, **y** stand for carbonyl carbon atoms.

Remarkably, the potency of glycopeptide antibiotics against *B. subtilis* ATCC 6633 was tested in ”competition” experiments in the presence of increasing amounts of (*N*-Ac)_2_-l-Lys-d-Ala-d-Ala [25]. Eremomycin (Figure 1) and dechloroeremomycin showed increasing activity in contrast to weakly dimerizing antibiotics such as vancomycin. This observation was explained with the ligand-enhanced dimerization mode (co-operativity) [13,26].

Using an ^15^N-labelled glycopeptide antibiotic for the first time [27], we proved the dynamic equivalence of the two sides of the dimeric eremomycin and the flexibility of the residue-3 Asn sidechain. We have also shown that the amides of the binding pocket exhibit the highest amide exchange rate, which could be a manifestation of the water strip effect presumably due to 2–3 amide bond rotation. ^15^N-labelling of glycopeptides provides an excellent tool for establishing the oligomeric state via the measurement of their global reorientational correlation time from ^15^N relaxation. 

Eremomycin forms a very strong homo-dimer among vancomycin antibiotics, and cooperativity between its dimerisation and ligand binding has been proved [25]. There is a strong correlation between the dimerisation constant (K_dim_) and antibacterial activity (MIC) for many glycopeptide antibiotics [28]. Until now, glycopeptide antibiotic oligomers larger than dimers have never been detected in aqueous solution, except using the size-exclusion chromatography method in case of vancomycin [18]. 

In the present work, we explore the higher-order oligomeric states of eremomycin in aqueous solution using ^15^N-labelled and unlabelled forms bound to different cell wall analogue peptides. In addition, antimicrobial activities are also investigated.

## 2. Results

### 2.1. NMR Relaxation and Diffusion Evidence of Ligand-Dependent Oligomerisation of ^15^N-Labelled Eremomycin

In our earlier ^15^N NMR relaxation study [27] at 280 K, we determined the global correlation time of the dimeric ^15^N-eremomycin in acetate buffer, which was found to be 3.7 ns. The ^1^H-^15^N HSQC spectra allowed the separate NH assignments of the two halves of the asymmetric eremomycin dimer (K_dim_ = 3 × 10^6^/M) [26]. Furthermore, it was demonstrated that the addition of *N*-Ac-d-Ala-d-Ala could slightly enhance the activity of the antibiotic in vitro. 

**Figure 2:**
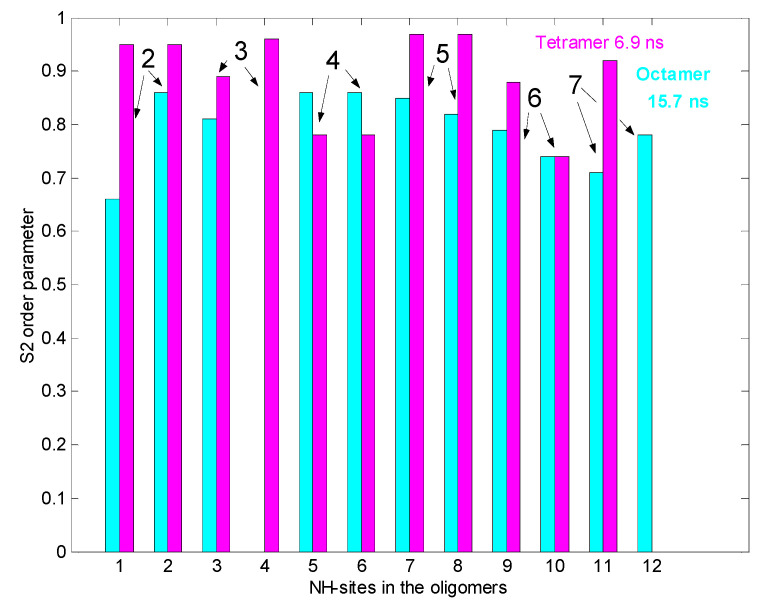
^15^NH S^2^ order parameters from ^15^N relaxation for tetra and octameric, *N*-Ac-d-Ala-d-Ala ligand-filled eremomycin (278 K). In the bar plot the tetramer is pink, and the octamer is cyan coloured. Numbers on the top of bars mean amino acid residues in the heptapeptide sequence.

**Figure 3:**
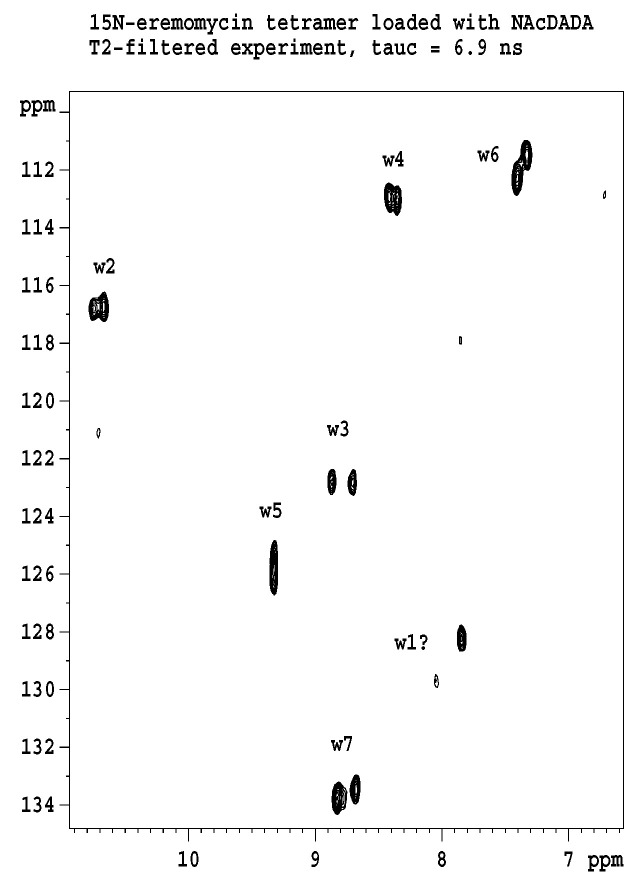
^15^N-T_2_ filtered HSQC experiment displays exclusively the tetramer signals, since the faster spin–spin relaxation destroys the signals of the octamer. Labels w stand for amide NH groups; numbers are according to sequential position. Signals are doubled because of the asymmetry of the dimeric building blocks. One of the w1 signals is too weak, and therefore is labelled with a question mark.

**Figure 4:**
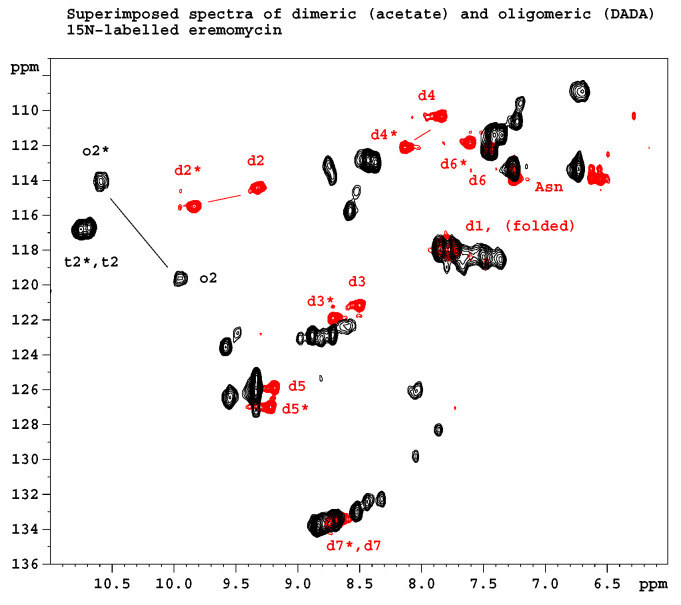
Overlayed ^15^N HSQC spectra of *N*-Ac-d-Ala-d-Ala ligand-filled oligomeric and dimeric eremomycin. Letters d, t and o stand for NH amide groups in dimers, tetramers and octamers, respectively. Numbers denote sequential order. Asterisks (*) label amide NH groups belonging to the same monomeric unit within an asymmetric dimer building block. Not all signals are labeled because of signal overlap.

Figure 2 shows the results of ^15^N relaxation experiments (T_1_, T_2_, ^15^N-^1^H NOE) [29] followed by the Lipari-Szabo model-free analysis [30,31] as applied to resolved NH signals, yielding S^2^ order parameters and reorientational correlation times in the tetra- and octamers. The global correlation time is roughly doubled in the octamers (15.7 ns vs. 6.9 ns) if compared to tetramers (A similar factor of two is found between tetramers and dimers). This is a reliable evidence of the presence of two kinds of oligomers (tetra and octa) parallel in the solution. Applying a ^15^N-T_2_ relaxation filter, the octamer signals can be removed from the ^15^N-HSQC spectra (Figure 3) that helped the signal assignments. The spectra of the oligomers overlayed with the dimer **(Figure 4**) clearly show twin signals for each form according to dominant back-to-back dimer constituents, with asymmetry due to disaccharide conformations [9]. The presence of putative ligand mediated face-to-face arrangements in oligomers did not change the basic asymmetry, as proven by doubled NMR signals throughout all oligomeric spectra. 

### 2.2. Titration of Eremomycin with N-Ac-d-Ala-d-Ala and N-Ac-d-Ala

**Figure 5:**
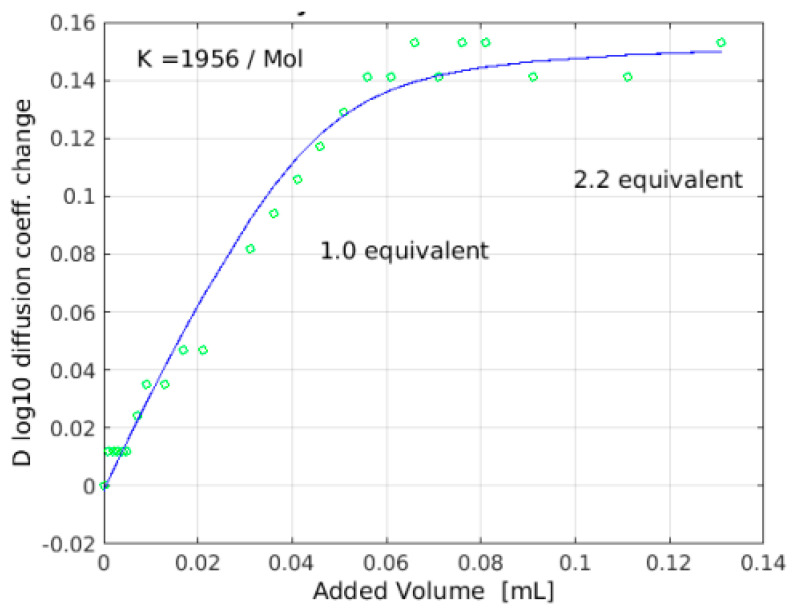
Titration of *N*-Ac-d-Ala-d-Ala into eremomycin as monitored by NMR diffusion constants (DOSY). Though the change of diffusion coefficient is negative on the y axis, they are shown as positive values for convenience of fitting.

**Figure 6:**
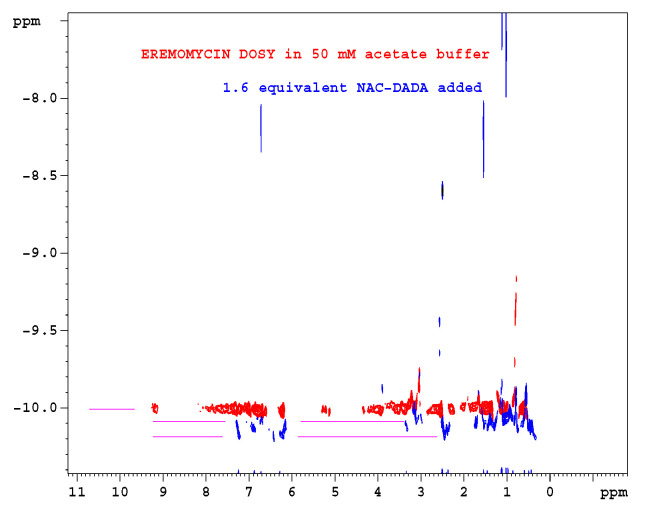
Overlayed NMR diffusion (DOSY) experiments of eremomycin: red is in the absence of ligand; blue is after coaddition of 1.6 equivalent *N*-Ac-d-Ala-d-Ala. On the log10-based vertical scale, a 0.1 unit difference indicates a factor of two for the molecular mass.

**Figure 7:**
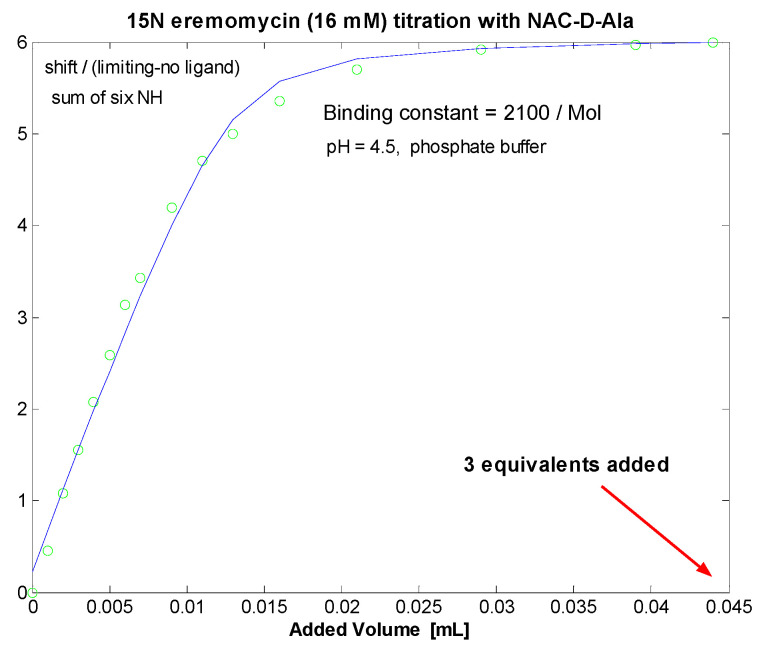
Chemical shift titration of dimeric ^15^N-labelled eremomycin with *N*-Ac-d-Ala.

**Figure 8:**
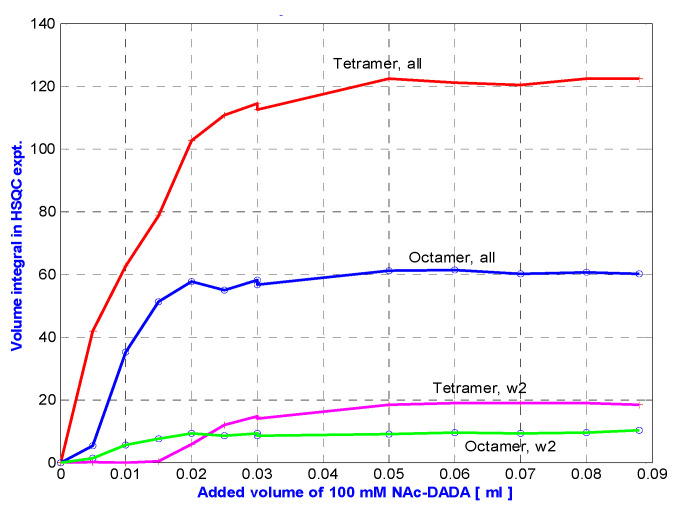
N-Ac-d-Ala-d-Ala titration into eremomycin solution at 278 K, followed by measuring the integral volumes of tetra and octameric NH signals.

Titration of 1-^13^C *N*-Ac-d-Ala-d-Ala [32] into 16 mM eremomycin solution was monitored measuring ^15^N-HSQC spectra and by observing the ^13^C NMR spectra of the ligand. Saturation of eremomycin with the dipeptide ligand was proven at the end of titration in accordance with published bulk binding constants (2800/M) [33], which means > 95% occupancy with the dipeptide ligand. Using the DOSY NMR titration technique (Figure 5 and Figure 6), we obtained a similar ligand binding affinity (K_e_ = 1960/Mol). *N*-Ac-d-Ala showed moderate binding to eremomycin in chemical shift titration experiments (Figure 7, Ke = 2100/Mol), similar to the dipeptide affinity. Titration of eremomycin with *N*-Ac-d-Ala-d-Ala was followed by ^15^N HSQC experiments (Figure 8).

**Figure 9:**
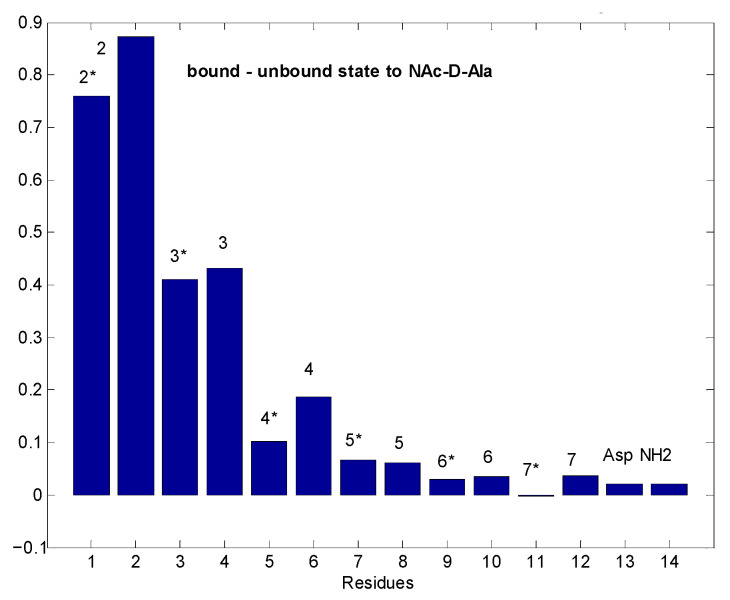
Differences in water saturation efficiency between the free eremomycin dimer and *N*-Ac-d-Ala loaded dimer. Bars close to 1 unit mean huge differences in water accessibility due to ligand binding, while values close to 0 mean no change in water access to that site upon binding. Asterisks indicate the same monomeric units within dimers. Numbers on the top of bars mean residue numbers, according to the scheme of Figure 1.

Figure 8 shows that in toto, ca. two times more tetramers are generated than octamers during *N*-Ac-d-Ala-d-Ala titration of eremomycin. However, exclusive observation of w2 signals might be misleading, suggesting that octamers are formed first. This effect may be due to water saturation impacts at the w2 site, which is the most sensitive, being close to the binding site. Solvent accessibility of the possible binding sites can be tracked from water saturation difference experiments (Figure 9) that locate the binding sites. 

**Figure 10:**
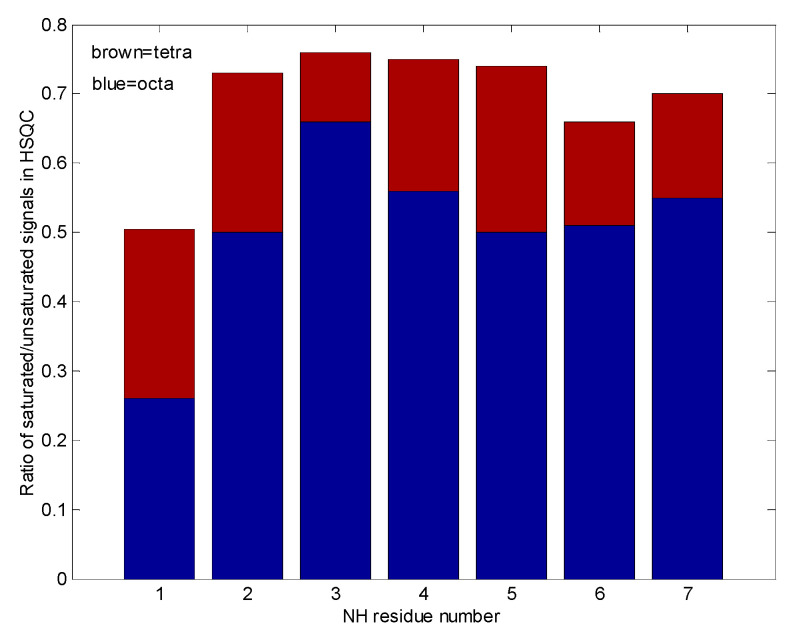
In this “pile up” bar graph, the tetra and octameric eremomycin water saturation efficiencies are compared. The smaller the contribution, the higher the probability of solvent access to that site.

Figure 9 shows that in dimeric eremomycin, there is a large difference in solvent accessibility between *N*-Ac-d-Ala-filled and free eremomycin dimers. Upon *N*-Ac-d-Ala binding to eremomycin, water is expelled from the binding pocket, suggesting that the binding site for *N*-Ac-d-Ala is around the w2, w3 amide groups. In contrast, solvent accessibility did not change far from the binding site. We have shown earlier in similar experiments [27] that the highest saturation is achieved around the binding site. If we compare in straight water saturation transfer experiments the tetramer and octamer mixture (Figure 10), we observe differences between them.

**Figure 11:**
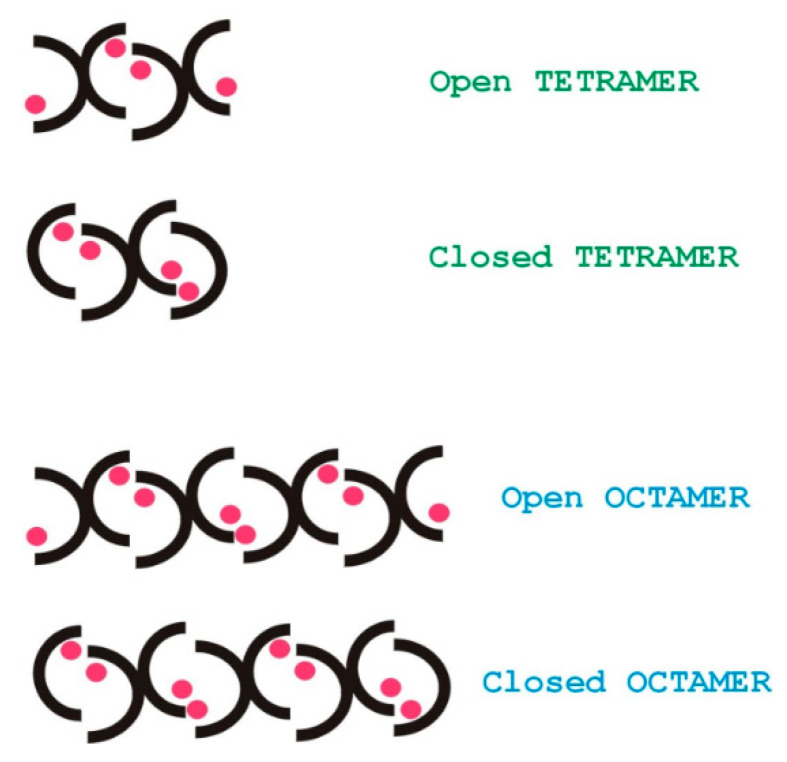
Schematic arrangement of the oligomers in open or closed conformations. Pink spots represent the ligands that can help to form face-to-face interfaces between glycopeptide monomers.

**Figure 12:**
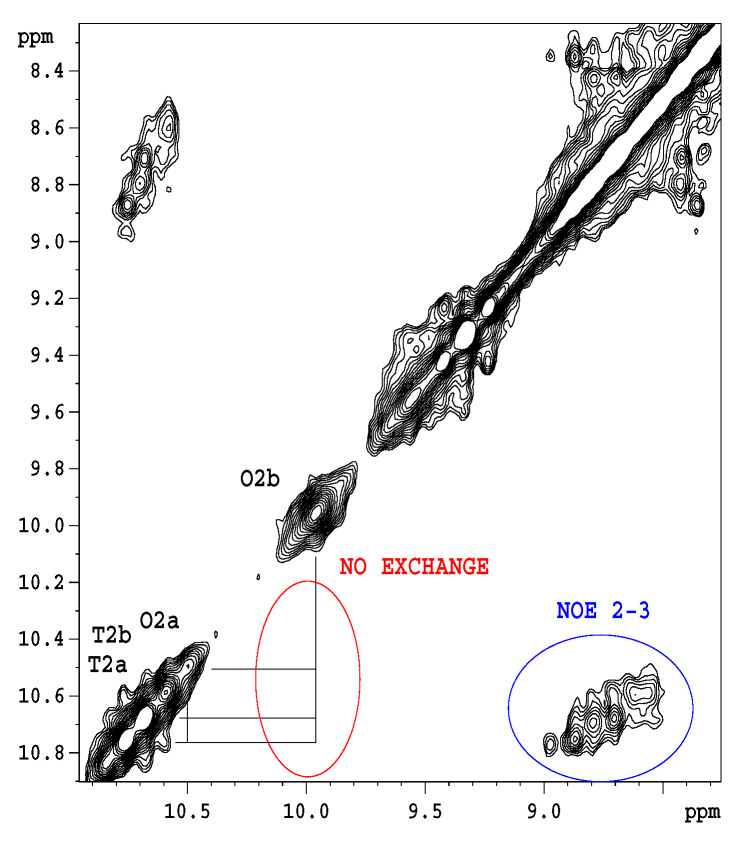
EXSY (NOESY) spectrum of 1-^13^C *N*-Ac-d-Ala-d-Ala-saturated eremomycin at 278 K, mixing time 100 ms. The w2 amide group signals belonging to tetramer (T2a, T2b) and octamer (O2a, O2b) are separated on the diagonal, but no off-diagonal exchange peaks are visible between tetramer and octamer states. However, the intramolecular NOESY peaks are observable.

Figure 10 shows qualitatively, that water accessibility is decreased in the octamers (the bottom blue bars) with respect to the tetramers (top brown bars) possibly because of more closed structures, that are already filled with ligands. The w3 amide group around the tetramer binding site has the highest chance for water access. Figure 11. explains the scheme for the arrangement of oligomers in "open" and "closed" conformations. We were curious to know how fast the interconversion between the tetra and octamers can happen. To learn more about the possible time scale of conversion, EXSY spectra were measured (Figure 12).

The EXSY spectrum (Figure 12) demonstrates the lack of chemical exchange between the octamer and tetramer on the slow NMR timescale (ms-s). Since during titrations no detectable chemical shift movements were observed, we can exclude fast exchange as well. Hence, octamers and tetramers persist simultaneously in solution, without fast exchange of their oligomeric state. 

### 2.3. Results of Antimicrobial Tests

#### 2.3.1. Checkerboard Method

The impact of the ligand *N*-Ac-d-Ala-d-Ala on the glycopeptide activity is shown in Table 1.

#### 2.3.2. Disk-Diffusion Method

We found that the relative potential depends on the strain selected. For *staphylococci*, the values were −20% (vancomycin), +20% (eremomycin), regardless of the concentration of glycopeptides and the ligand. Extreme values were found and determined for *Enterococcus faecalis* 9 (Table 2).

## 3. Discussion

Evidence for ligand-induced eremomycin oligomerization has been found in aqueous solution for the first time, based on NMR ^1^H-^15^N HSQC spectra, chemical shift titration, relaxation, and diffusion measurements. Dimeric eremomycin building blocks oligomerize to form discrete octamers and tetramers in the presence of increasing amounts of *N*-Ac-d-Ala-d-Ala. Dimers were exclusively observed in the presence of similar ligands terminating in the d-Ala sequence. In addition, we demonstrate that increasing ligand size diminishes or even stops the breathing flip-flop motion of the ring-4 disaccharide that overhangs the binding site, which suggests that exchange due to such motion may happen mainly intramolecularly, without breaking the dimeric structures. It is well known that glycopeptide antibiotics form non-covalent, head-to-tail dimers in aqueous solution with a kind of antiparallel β-strand arrangement of the peptide backbone. In solution NMR, there is a fingerprint indicator of dimerisation, an unusual ^1^H NMR chemical shift of an aromatic proton signal, due to an orthogonal σ-π interaction of 6e (CH) hydrogen, exhibiting a proton chemical shift around 5 ppm, while the ^13^C shift of 6e-C is in the common aromatic range, at ca. 122 ppm (Figure S1). This effect is always observed in aqueous solutions and is never seen in DMSO solution. Perhaps the lack of water is the reason why dimers were not observed by solid-state NMR, where glycopeptides were investigated using bacterial cell wall lysates, applying trehalose for water replacement. Using X-ray crystallography, higher-order oligomers of dimer replicas were often detected and structurally characterized, proving that ligands in the binding sites generate contiguous back-to-back and face-to-face dimers, leading to supramolecular structures. In this work we identified *N*-Ac-d-Ala-d-Ala-induced eremomycin tetramers and octamers in aqueous solutions, closer to in vivo conditions. In the case of vancomycin, the low dimerization constant and the high affinity to the ligand allow oligomer formations possibly by breaking the dimeric structures. Then, many concurrent oligomers may be formed simultaneously in exchange with each other, hindering NMR observation and characterisation. Still, we attempted to observe if higher-order vancomycin oligomers are formed in aqueous solution upon *N*-Ac-d-Ala-d-Ala titration. At 280 K, the NMR DOSY experiments displayed a blurred, overlapping diffusion front (not shown), that accords to an average mass around hexamers in the presence of one equivalent ligand. Increasing the temperature to 320 K slightly decreased the average oligomer size. However, it is not yet clear if monomers, dimers, and/or higher-order oligomers of glycopeptide antibiotics persist in vivo, in clinically applied concentrations [34]. 

**Figure 13:**
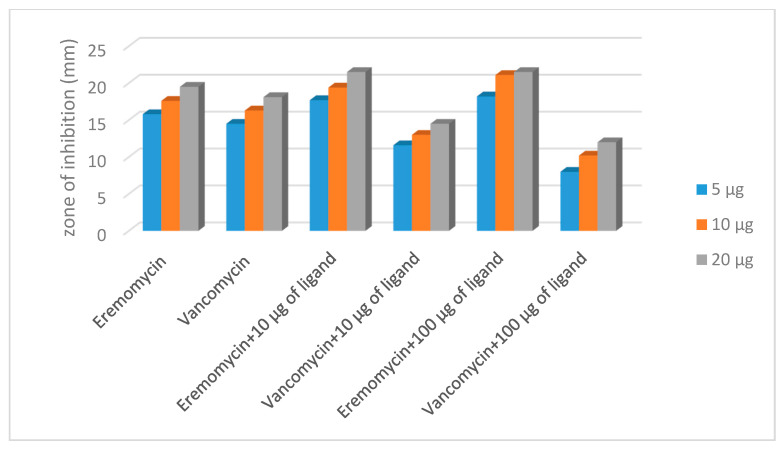
The change in the diameter zone inhibition in the presence antibiotics alone and in interaction with *N*-Ac-d-Ala-d-Ala (*S. aureus* 209P).

Influence of the presence of the cell-wall analogue peptide *N*-Ac-d-Ala-d-Ala ligand with respect to the antimicrobial activity of glycopeptide antibiotics vancomycin and eremomycin was evaluated by checkboard and disk diffusion methods. The obtained results are presented in Table 1 and Table 2. The results obtained by the checkboard method revealed the following tendency of changes in antimicrobial activity: the decrease of the antibiotic/ligand ratio resulted in a decrease in the MIC values for eremomycin for all strains, while the MIC values for vancomycin in the presence of peptide ligand either did not change or increased (2xMIC) for *S. aureus* 20450. A significant difference in the interaction of *N*-Ac-d-Ala-d-Ala and vancomycin or eremomycin was observed in the agar-diffusion assay (Table 2, Figure 13). Changes in the concentration of eremomycin and ligand did not affect the relative potential—the zones of inhibition for three strains of test microorganisms increased in the same way as when the antibiotic was combined with *N*-Ac-d-Ala-d-Ala. When exposed to vancomycin, the effect was microorganism-dependent. For *staphylococci*, the values were −20%, and as a function of the concentration of vancomycin in combination with the ligand, the diffusion zones decreased proportionally. The extreme values were obtained for *Enterococcus faecalis 9*. In the presence of 5 µg/mL *N*-Ac-d-Ala-d-Ala, the zones of inhibition changed the concentration dynamics; the relative potential decreased with increasing antibiotic concentration (Figure 13).

## 4. Materials and Methods 

### 4.1. NMR Spectroscopy

A Bruker Avance-II NMR spectrometer (500.13 MHz ^1^H and 50.68 MHz ^15^N frequency) equipped with a multinuclear bbi z-gradient probehead was used for all measurements either at 298 K or 278 K temperature. Diffusion experiments were carried out using Bruker’s ‘ledbpgs2s’ stimulated echo DOSY pulse sequence including bipolar and spoil gradients. This was extended with a 2 × 4 ms spin echo period before the detection period to suppress the baseline shift [35]. ^15^N relaxation experiments were carried out according to [29] and evaluated with the model-free approach [30,31] using the isotropic rotational diffusion approach and a single global correlation time. The recycle delays in T_1_, T_2_ experiments were typically 2.5 s, but 5 s in heteronuclear ^15^N-(^1^H) NOE. In water saturation difference experiments, two ^15^N-^1^H HSQC experiments were run, using 3 s selective water presaturation in one of them, and the difference was calculated by comparison with an undisturbed reference.

### 4.2. Antibiotics and Other Reagents

*N*-Ac-d-Ala-d-Ala was obtained from NovaBioChem (Merck Group, Darmstadt, Germany), vancomycin was obtained from Merck (Darmstadt, Germany), while eremomycin was prepared in the Gause Institute of new Antibiotics (Moscow, Russia). ^15^N labelled eremomycin and 1-^13^C *N*-Ac-d-Ala-d-Ala were produced as described earlier [27,32]. 

### 4.3. Bacterial Strains

Methicillin-resistant *Staphylococcus aureus* (MRSA) strain 20450, vancomycin–resistant *Enterococcus faecalis 9,* and *Staphylococcus aureus 209P (ATCC 6538P)* were obtained from the Medical Microbiology Laboratory of State Research Center for Antibiotics (Moscow, Russia).

### 4.4. Determination of Antibiotic Minimum Inhibitory Concentration

Evaluation of Minimum Inhibitory Concentrations (MIC, μg/mL) of vancomycin and eremomycin was carried out using the microdilution method in Mueller–Hinton broth. For MIC determination, 96-well microtiter plates, containing a dilution series of the antibiotics, were inoculated with 10^5^ CFU/mL and incubated at 37 °C for 24 h.

A range of antibiotic concentrations based on the obtained MIC values was selected to analyze the interaction of glycopeptides with *N*-Ac-d-Ala-d-Ala.

### 4.5. Interaction of Glycopeptides and the Ligand

#### 4.5.1. Checkerboard Method

A range of antibiotic concentrations based on the obtained MIC values was selected to analyze the interaction of glycopeptides with *N*-Ac-d-Ala-d-Ala. Interactions between glycopeptides and dipeptide were investigated in 96-well microtiter plates in the Mueller–Hinton broth. The inoculum contained 10^5^ CFU/mL. The MIC of the glycopeptides (μM) for each concentration of ligand was read 18 h after incubation at 37 °C. 

Microtiter plates with the antibiotic and peptide ligand were prepared either in advance and kept at 4 °C for 18 h or prepared immediately before inoculum introduction.

#### 4.5.2. Disk-Diffusion Method 

The standard 6 mm discs to which 20 μL of a solution containing 10 or 100 μg of *N*-Ac-*d*-Ala-*d*-Ala in H_2_O (0.5 or 5 mg/mL) was applied were dried in a laminar flow at room temperature. Then, the solution of the antibiotic was added and the disks were dried again. Glycopeptide concentrations were 5, 10, and 20 μg per disc. The disks were placed on agar medium, containing 10^8^ CFU/mL of microorganism strain. The discs loaded with glycopeptide antibiotics solely were used to compare the zones of inhibition. Zones of inhibition were measured after 18–24 h of incubation at 37 °C. The diameters of the zones of inhibition were measured with a caliper, and the average of nine values for each experimental point was calculated. The relative potential was determined by dividing the larger diameter by the smaller one. The sign (–) corresponds to a decrease, and a positive value to an increase in the zone of inhibition of the antibiotic *N*-Ac-*d*-Ala-*d*-Ala.

## 5. Conclusions

Higher-order glycopeptide antibiotic oligomers, first suggested by crystal studies, are here shown to be formed in aqueous solution as well. However, in contrast to X-ray-detected vancomycin hexamers [18], we detected a mixture of tetra-and octameric eremomycin in solution, depending on the pertinent dipeptide ligand concentration. Interestingly, oligomerisation was induced exclusively by the dipeptide *N*-Ac-d-Ala-d-Ala. Neither *N*-Ac-d-Ala nor the cell wall analogue tripeptide (*N*-Ac)_2_-Lys-d-Ala-d-Ala induced higher-order oligomerisation. 

In any glycopeptide antibiotic, oligomeric-form NMR signal doubling is observed, characteristic of asymmetric back- to back dimer building blocks contributing to the pseudo-C2 symmetry of higher-order oligomers. It is not yet clear if such oligomers are also generated in vivo when glycopeptides are used for medical treatments, though slight increases in antibacterial effect could be induced by some model peptides. Synthetic, multivalent ligands provide a promising alternative to enhance ligand binding [36]. 

Ligand-induced oligomer formation of eremomycin slightly increased the antibacterial activity when the ligand concentration was low at the beginning, but at increased *N*-Ac-d-Ala-d-Ala concentrations, the competition reduced this beneficial effect. If eremomycin was loaded at a stoichiometrically relevant ligand quantity from the starting point, then adding more ligand in consecutive steps did not change the antimicrobial activity. The differences in antibacterial effect between vancomycin and eremomycin in the presence of *N*-Ac-d-Ala-d-Ala may result from the differences in their mode of action affecting transpeptidation and transglycosylation.

Specifically, ligand-induced and -mediated reversible oligomerization of glycopeptide antibiotics is an interesting example of supramolecular structures, and formation of ligand-controlled peptide self-assemblies is challenging in their own right, which might even have a distant analogy with amyloid plaque formation [37]. Reprogramming or modulating the antimicrobial effects in vancomycin-type antibiotics with covalent modifications might be promising, since some glycopeptides were shown to have antiviral effects [38].

## Figures and Tables

**Table 1:** Antibacterial activities of glycopeptides as influenced by the *N*-Ac-d-Ala-d-Ala dipeptide ligand (checkerboard method).

Microorganisms	Glycopeptide	Concentration of Ligand (μM/mL)								
		0	8	16	32	64	128	256	512	1024
		Glycopeptide MIC (μM/mL)								
*S. aureus* 20450	vancomycin	1	2	2	2	2	2	2	4	8
	eremomycin	0.12	0.06	0.06	0.06	0.06	0.125	0.125	0.125	0.5
*E. faecalis* 9	vancomycin	16	16	16	16	16	16	32	32	64
	eremomycin	0.25	0.12	0.25	0.25	0.25	0.25	0.25	0.5	1
*S. aureus* 209P	vancomycin	1	1	1	1	1	1	2	2	4
	eremomycin	0.06	0.03	0.03	0.03	0.03	0.03	0.03	0.06	0.125

**Table 2:** Antibacterial activities of glycopeptides as influenced by the *N*-Ac-d-Ala-d-Ala dipeptide ligand (disk diffusion method) and relative potency against the *Enterococcus faecalis* 9 strain.

*N*-Ac-d-Ala-d-Ala (μg/mL)	0			10			100		
Glycopeptide Concentration (μg/mL)	5	10	20	5	10	20	5	10	20
Vancomycin	**diffusion zones (mm)**								
	14.5	16.3	18.1	11.6	13	14.5	8	10.2	12
	**relative potency**								
	-			−25%	−25%	−25%	−80%	−60%	−20%
Eremomycin	**diffusion zones (mm)**								
	15.8	17.6	19.5	17.7	19.4	21.5	18.2	21.1	21.5
	**relative potency**								
	-			+12%	+10%	+10%	+15%	+20%	+10%

## Data Availability

More data of this work are available in supplementary material.

## Supplementary Materials

The following are available online at https://www.mdpi.com/1424-8247/14/2/83/s1, Figure S1: ^1^H-^13^C HSQC spectrum of the eremomycin dimer at 298 K, at pH 4.5 in 20 mM acetate (D_2_O) buffer (Bruker NEO-700 NMR spectrometer, equipped with Prodigy, TCI probehead). Materials and methods for antimicrobial tests with N-Ac-D-Ala-D-Ala-loaded eremomycin.

## References

[1] Healy V.L., Lessard I.A.D., Roper D.I., Knox J.R., Walsh C.T. (2000). Vancomycin resistance in enterococci: Reprogramming of the D-Ala-D-Ala ligases in bacterial peptidoglycan biosynthesis. Chem. Biol..

[2] Reynolds P.E., Courvalin P. (2005). Vancomycin resistance in enterococci due to synthesis of precursors terminating in D-Alanyl-D-Serine. Antimicrob. Agents Chemother..

[3] Zeng D., Debabov D., Hartsell T.L., Cano R.J., Adams S., Schuyler J.A., McMillan R., Pace J.L. (2016). Approved Glycopeptide Antibacterial Drugs: Mechanism of Action and Resistance. Cold Spring Harb. Perspect. Med..

[4] Blaskovich M.A.T., Hansford K.A., Butler M.S., Jia Z.G., Mark A.E., Cooper M.A. (2018). Developments in Glycopeptide Antibiotics. Acs Infect. Dis..

[5] Loll P.J., Axelsen P.H. (2000). The structural biology of molecular recognition by vancomycin. Annu. Rev. Biophys. Biomol. Struct..

[6] Waltho J.P., Williams D.H. (1989). Aspects of Molecular Recognition - Solvent Exclusion and Dimerization of the Antibiotic Ristocetin When Bound to a Model Bacterial Cell-Wall Precursor. J. Am. Chem. Soc..

[7] Batta G., Sztaricskai F., Kover K.E., Rudel C., Berdnikova T.F. (1991). An NMR Study of Eremomycin and its Derivatives Full H-1 and C-13 Assignment, Motional Behavior, Dimerization and Complexation with Ac-D-ALA-D-ALA. J. Antibiot..

[8] Gerhard U., Mackay J.P., Maplestone R.A., Williams D.H. (1993). The Role of the Sugar and Chlorine Substituents in the Dimerization of Vancomycin Antibiotics. J. Am. Chem. Soc..

[9] Groves P., Searle M.S., Mackay J.P., Williams D.H. (1994). The Structure of an Asymmetric Dimer Relevant to the Mode of Action of the Glycopeptide Antibiotics. Structure.

[10] Beauregard D.A., Williams D.H., Gwynn M.N., Knowles D.J.C. (1995). Dimerization and Membrane Anchors in Extracellular Targeting of Vancomycin Group Antibiotics. Antimicrob. Agents Chemother..

[11] Batta G., Cristofaro M.F., Sharman G.J., Williams D.H. (1996). Demonstration of the difference in binding affinity between the two binding sites of the ristocetin A asymmetric dimer. Chem. Commun..

[12] Bardsley B., Williams D.H. (1997). Measurement of the different affinities of the two halves of glycopeptide dimers for acetate. Chem. Commun..

[13] Shiozawa H., Chia B.C.S., Davies N.L., Zerella R., Williams D.H. (2002). Cooperative binding interactions of glycopeptide antibiotics. J. Am. Chem. Soc..

[14] McPhail D., Cooper A. (1997). Thermodynamics and kinetics of dissociation of ligand-induced dimers of vancomycin antibiotics. J. Chem. Soc.-Faraday Trans..

[15] Linsdell H., Toiron C., Bruix M., Rivas G., Menendez M. (1996). Dimerization of A82846B, vancomycin and ristocetin: Influence on antibiotic complexation with cell wall model peptides. J. Antibiot..

[16] Loll P.J., Miller R., Weeks C.M., Axelsen P.H. (1998). A ligand-mediated dimerization mode for vancomycin. Chem. Biol..

[17] Schafer M., Sheldrick G.M., Schneider T.R., Vertesy L. (1998). Structure of balhimycin and its complex with solvent molecules. Acta Crystallogr. Sect. D Biol. Crystallogr..

[18] Loll P.J., Derhovanessian A., Shapovalov M.V., Kaplan J., Yang L., Axelsen P.H. (2009). Vancomycin Forms Ligand-Mediated Supramolecular Complexes. J. Mol. Biol..

[19] Jia Z.G., O’Mara M.L., Zuegg J., Cooper M.A., Mark A.E. (2013). Vancomycin: Ligand recognition, dimerization and super-complex formation. FEBS J..

[20] Loll P.J., Bevivino A.E., Korty B.D., Axelsen P.H. (1997). Simultaneous recognition of a carboxylate-containing ligand and an intramolecular surrogate ligand in the crystal structure of an asymmetric vancomycin dimer. J. Am. Chem. Soc..

[21] Lehmann C., Bunkoczi G., Vertesy L., Sheldrick G.M. (2002). Structures of glycopeptide antibiotics with peptides that model bacterial cell-wall precursors. J. Mol. Biol..

[22] Cooper M.A., Williams D.H. (1999). Binding of glycopeptide antibiotics to a model of a vancomycin-resistant bacterium. Chem. Biol..

[23] Cooper M.A., Williams D.H., Cho Y.R. (1997). Surface plasmon resonance analysis of glycopeptide antibiotic activity at a model membrane surface. Chem. Commun..

[24] Good V.M., Gwynn M.N., Knowles D.J.C. (1990). MM-45289, A Potent Glycopeptide Antibiotic which Interacts Weakly with Diacetyl-L-Lysyl-D-Alanyl-D-Alanine. J. Antibiot..

[25] Beauregard D.A., Maguire A.J., Williams D.H., Reynolds P.E. (1997). Semiquantitation of cooperativity in binding of vancomycin-group antibiotics to vancomycin-susceptible and -resistant organisms. Antimicrob. Agents Chemother..

[26] Mackay J.P., Gerhard U., Beauregard D.A., Westwell M.S., Searle M.S., Williams D.H. (1994). Glycopeptide Antibiotic-Activity and the Possible Role of Dimerization—A Model for Biological Signaling. J. Am. Chem. Soc..

[27] Batta G., Sztaricskai F., Makarova M.O., Gladkikh E.G., Pogozheva V.V., Berdnikova T.F. (2001). Backbone dynamics and amide proton exchange at the two sides of the eremomycin dimer by N-15 NMR. Chem. Commun..

[28] Allen N.E., Nicas T.I. (2003). Mechanism of action of oritavancin and related glycopeptide antibiotics. FEMS Microbiol. Rev..

[29] Farrow N.A., Muhandiram R., Singer A.U., Pascal S.M., Kay C.M., Gish G., Shoelson S.E., Pawson T., Formankay J.D., Kay L.E. (1994). Backbone Dynamics of a Free and a Phosphopeptide -Complexed SRC Homology-2 Domain Studied by N-15 NMR Relaxation. Biochemistry.

[30] Lipari G., Szabo A. (1982). Model-Free Approach to the Interpretation of Nuclear Magnetic-Resonance Relaxation in Macromolecules. 1. Theory and Range of Validity. J. Am. Chem. Soc..

[31] Lipari G., Szabo A. (1982). Model-Free Approach to the Interpretation of Nuclear Magnetic-Resonance Relaxation in Macromolecules. 2. Analysis of Experimental Results. J. Am. Chem. Soc..

[32] Batta G., Kover K.E., Szekely Z., Sztaricskai F. (1992). Glycopeptide Binding-Site Spied through Transferred Heteronuclear NOE - 1-C-13 Ac-D-Ala-D-Ala Bonded to Vancomycin and Ristocetin-A. J. Am. Chem. Soc..

[33] Sharman G.J., Searle M.S., Benhamu B., Groves P., Williams D.H. (1995). Burial of Hydrocarbon Causes Cooperative Enhancement of Electrostatic Binding. Angew. Chem. Int. Ed. Eng..

[34] Phillips-Jones M.K., Lithgo R., Dinu V., Gillis R.B., Harding J.E., Adams G.G., Harding S.E. (2017). Full hydrodynamic reversibility of the weak dimerization of vancomycin and elucidation of its interaction with VanS monomers at clinical concentration. Sci. Rep..

[35] Orosz L., Batta G., Keki S., Nagy M., Deak G., Zsuga M. (2007). Self-association of bis-(alpha,beta-D-glucopyranosyl)-polyisobutylene. Carbohydr. Res..

[36] Nicolaou K.C., Boddy C.N.C., Brase S., Winssinger N. (1999). Chemistry, biology, and medicine of the glycopeptide antibiotics. Angew. Chem.-Int. Ed..

[37] Iadanza M.G., Jackson M.P., Hewitt E.W., Ranson N.A., Radford S.E. (2018). A new era for understanding amyloid structures and disease. Nat. Rev. Mol. Cell Biol..

[38] Szűcs Z., Naesens L., Stevaert A., Ostorházi E., Batta G., Herczegh P., Borbás A. (2020). Reprogramming of the Antibacterial Drug Vancomycin Results in Potent Antiviral Agents Devoid of Antibacterial Activity. Pharmaceuticals.

