# Supplementary material for: Bacterial Cell Wall Analogue Peptides Control the Oligomeric States and Activity of the Glycopeptide Antibiotic Eremomycin: Solution NMR and Antimicrobial Studies
Source: Pharmaceuticals (Basel). 2021 Jan 22;14(2):83. doi: 10.3390/ph14020083 (PMC7911593; doi:10.3390/ph14020083)
Supplement: Supplementary file 1 [file pharmaceuticals-14-00083-s001.pdf]

Bacterial cell wall analogue peptides control the oligomeric states and activity of the glycopeptide antibiotic eremomycin. Solution NMR and antimicrobial studies.

László Izsépi,[1,2] Réka Erdei,[2] Anna N. Tevyashova,[3] Natalia E. Grammatikova, [3] Andrey E. Shchekotikhin ,[3] Pál Herczegh[4] and Gyula Batta[2*]

[1] Doctoral School of Chemistry, University of Debrecen, H-4032 Debrecen, Egyetem tér 1., Hungary; izsepi.laszlo@gmail.com (L.I.)
[2] Department of Organic Chemistry, University of Debrecen, H-4032 Debrecen, Egyetem tér 1., Hungary; rerdeii@yahoo.com (R.E.)
[3] Gause Institute of New Antibiotics, 11 B. Pirogovskaya, Moscow, 119021, Russia; chulis@mail.ru, (A.T.) shchekotikhin@mail.ru, (A.E.S.) ngrammatikova@yandex.ru, (N.E.G.)
[4] Department of Pharmaceutical Chemistry, University of Debrecen, H-4032 Debrecen, Egyetem tér 1., Hungary; herczegh.pal@pharm.unideb.hu (P.H.)
[2*] Correspondence: batta@unideb.hu (G.B.);

Supplementary material:

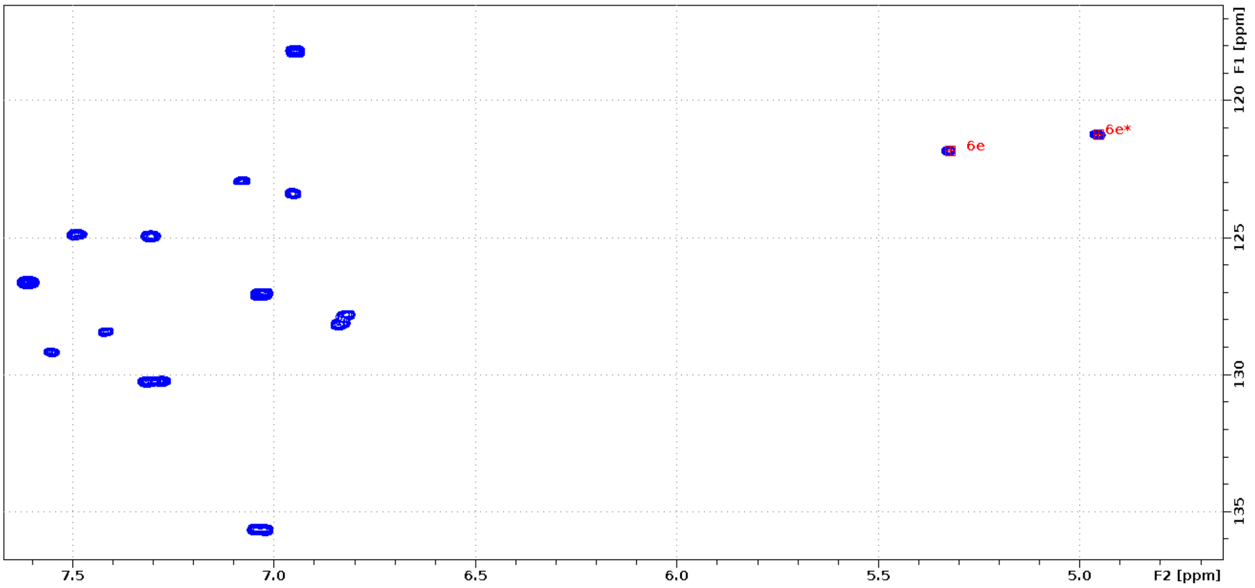

Figure S1. ^{1}H-^{13}C HSQC spectrum of the eremomycin dimer at 298K, at pH 4.5 in 20 mM acetate (D_2O) buffer (Bruker NEO-700 NMR spectrometer, equipped with Prodigy, TCI probehead) . Signal doubling in the aromatic region is observed, and the strongest impact is seen for the 6e signals, possessing unusual ~ 5 ppm ^{1}H chemical shifts for aromatic CH groups, but characteristic for glycopeptide dimers.

<u>Materials and methods for antimicrobial tests:</u>

<u>Antibiotics and Other Reagents</u>

N-Acetyl-D-alanyl-D-alanine (N-Ac-D-Ala-D-Ala) was obtained from NovaBioChem (Switzerland), Vancomycin was obtained from ICN Biomedicals (Ohio, USA), Eremomycin was obtained from Gause Research Institute of New Antibiotics (Moscow, Russia).

Bacterial strains

Methicillin-resistant *Staphylococcus aureu*s strain 20450 (MRSA), vancomycin–resistant *Enterococcus faecalis* 9 and *Staphylococcus aureus* 209P (ATCC 6538P) were obtained from the Medical Microbiology Laboratory of State Research Center for Antibiotics (Moscow, Russia).

The characteristics of the *E.faecalis 9* strain are presented in the table below.

MIC *E.faecalis 9* (μg/ml)		
Vancomycin	Teicoplanin	Van B
32	1	

Preparation of experimental solutions

Starting stock solutions (μM) () were prepared in the Muller Hinton nutrient medium in the following concentrations:

N-Acetyl-D-alanyl-D-alanine 2; 4; 6; 32; 64; 96; 100; 200 μM

Eremomycin – 2; 32 μM

Starting stock solutions of antibiotic and peptide ligand were mixed 1:1 (v:v) to obtain the mixed stock solutions with the following concentrations of each component:

Mixed stock solution concentration obtained, μM		Ratio
N-Ac-D-Ala-D-Ala	Eremomycin	Ligand:antibiotic
1	1	1:1
2	1	2:1
3	1	3:1
50	1	50:1
100	1	100:1
16	16	1:1
32	16	2:1
48	16	3:1

Before determining the antimicrobial activity, the experimental solutions were incubated at room temperature overnight.

Determination of Antibiotic Minimum Inhibitory Concentration

Evaluation of Minimum Inhibitory Concentrations (MIC, μM/ml) of N-Ac-D-Ala-D-Ala:Eremomycin mixture was carried out using microdilution method in 96-wells microtiter plates in the Mueller-Hinton broth. The inoculum contained 10^5 CFU/ml. The MIC of the ligand:glycopeptide mixture for each concentration was determined after 18 hours of incubation at 37°C.

Results of MIC testing:

Strain	MIC, μM/ml					
	Eremo	1:1	1:2	1:3	1:50	1:100
S.aureus 209P	0.125	0.125	0.125	0.125	0.125	0.125
S.aureus 20450 (MRSA)	0.125	0.125	0.125	0.125	0.125	0.125

E.faecalis 9	0.5	0.5	0.5	0.5	0.5	0.5

The minimum inhibitory concentration of Eremomycin in all studied ratios of Eremomycin and N-Ac-D-Ala-D-Ala (from 1:1 to 100:1 ligand:antibiotic ratio) did not change.

In contrast to this experience, previous analysis showed that in the ratio of Eremomycin to ligand 1: 1000 - 1: 4000/8000 antimicrobial activity decreased by 2 dilutions for *Enterococcus faecalis* 9 and two strains of *S. aureus* respectively (please, see previous results - Checkerboard method).
